# The paradox of the evidence about invasive fungal infection prevention

**DOI:** 10.1186/s13054-016-1284-7

**Published:** 2016-04-27

**Authors:** Andrea Cortegiani, Vincenzo Russotto, Santi Maurizio Raineri, Antonino Giarratano

**Affiliations:** Department of Biopathology and Medical Biotechnologies (DIBIMED), Section of Anesthesia, Analgesia, Intensive Care and Emergency, Policlinico P. Giaccone, University of Palermo, Via del Vespro 129, 90127 Palermo, Italy

Invasive fungal infections (IFIs) are characterized by high morbidity and mortality in non-neutropenic critically ill patients. Attributable mortality due to *Candida* spp*.* infections ranges from about 42 to 63 % [1, 2]. Data from large observational and retrospective studies show an association between early antifungal treatment and improved survival [3, 4]. Updated clinical practice guidelines for the management of candidiasis have been recently published [5].

In 2006, Playford et al. published a Cochrane systematic review investigating the use of antifungal agents for prevention of IFIs in non-neutropenic critically ill patients [6]. In that review, the outcome of proven IFI was defined as a clinical illness consistent with the diagnosis and either histopathological evidence of IFI or a positive fungal culture from one or more sterile site specimens (including blood). Notably, funguria (as indicated by a positive urine fungal culture), in the absence of a complicated urinary tract infection, and fungal esophagitis were classified not as IFIs but as superficial fungal infections. The review included 12 studies and 1606 patients, and the use of antifungal agents was associated with a mortality reduction of about 25 % and with an IFI reduction of about 50 %. Recently, we updated the original review by Playford et al., including 22 randomized controlled trials (RCTs) and 2761 patients [7]. We modified the definition of the outcome-proven IFI excluding positive culture of *Candida* spp*.* from the respiratory tract, even in the presence of systemic or respiratory signs of infection, and classifying it as colonization instead of IFI. Untargeted antifungal treatment, encompassing prophylactic, pre-emptive, and empiric regimens [8], was not associated with a significant mortality reduction (moderate-quality evidence). However, antifungal agents reduced IFIs by about 45 % with low-grade-quality evidence. From these data, three clinical questions may arise.

## 1. How is it possible to observe a reduced invasive fungal infection without any significant survival benefit?

The original review included studies published before 2006 and only one multicenter study [9]. In the following years, several multicenter studies have been published. New molecules and approaches for untargeted antifungal treatment were tested but without any survival benefit [10–12]. The inclusion of multicenter studies improved the overall quality of available evidence, increased the total number of patients, and possibly diluted the original effect size on mortality. Additionally, it may be argued that the care of critically ill patients improved in terms of management and prevention of sepsis. This may have led to a blunted effect of the reduction of IFIs on mortality. Another possible explanation may rely on the recently described suppressive immunophenotype of septic patients with candidemia. From this perspective, it may be hypothesized that, although an effective antifungal treatment led to microbiological eradication and reduced fungal load and incidence of IFIs, patients still die from the consequences of their underlying impaired immunological function [13].

## 2. Is there an untargeted antifungal strategy or an antifungal drug more effective than others?

Subanalyses did not show any survival benefit from the use of either prophylactic or empiric treatment. No effect was detected for azoles, echinocandins, absorbable, or non-absorbable antifungals. Antifungal prophylaxis was associated with a significant reduction of IFI, whereas empiric treatment was not [7]. Only one RCT evaluated the pre-emptive approach and few patients were enrolled [14]. It may be argued that studies investigating empiric treatment, defined as the administration of antifungals in patients with signs/symptoms of infections at risk for IFIs, enrolled subjects with a more advanced disease process, leading to lack of efficacy. Azoles were associated with a significant reduction of IFIs, whereas studies investigating echinocandins did not show any significant benefit on IFI reduction. Notably, the numbers of studies and included patients were higher for the subanalyses of prophylaxis and azoles. An ongoing multicenter RCT will provide more data on the use of empiric treatment with echinocandins [15].

## 3. Should clinicians administer antifungals prior to definitive diagnosis of invasive fungal infection?

According to the available evidence from RCTs, untargeted antifungal therapy may lead to a reduction of IFIs without any survival benefit in non-neutropenic critically ill patients. Physicians should evaluate, case by case, the risks and benefits of the antifungal treatment after considering timing, risk factors, local microbiological epidemiology, and costs. Moreover, the extended use of untargeted antifungal treatment may be associated with increased resistance to these drugs [16]. Physicians should be aware that evidence from the last Cochrane review could not evaluate the relationship between severity of illness and potential benefit of antifungal treatment. There is a need for RCTs investigating the effectiveness of pre-emptive antifungal approaches (i.e., surrogate marker-driven treatment).

To solve the paradox, future studies should also better evaluate the pathophysiology of the IFI process in order to answer the challenging question of whether critically ill patients would die of or with IFIs.

## Abbreviations

IFI: invasive fungal infection
RCT: randomized controlled trial
